# Flagellar Genes Are Associated with the Colonization Persistence Phenotype of the Drosophila melanogaster Microbiota

**DOI:** 10.1128/spectrum.04585-22

**Published:** 2023-04-13

**Authors:** Sarah J. Morgan, John M. Chaston

**Affiliations:** a Plant and Wildlife Sciences, Brigham Young University, Provo, Utah, USA; Connecticut Agricultural Experiment Station

**Keywords:** colonization, drosophila, establishment, flagella, microbiome, microbiota, persistence

## Abstract

In this work, we use Drosophila melanogaster as a model to identify bacterial genes necessary for bacteria to colonize their hosts independent of the bulk flow of diet. Early work on this model system established that dietary replenishment drives the composition of the D. melanogaster gut microbiota, and subsequent research has shown that some bacterial strains can stably colonize, or persist within, the fly independent of dietary replenishment. Here, we reveal transposon insertions in specific bacterial genes that influence the bacterial colonization persistence phenotype by using a gene association approach. We initially established that different bacterial strains persist at various levels, independent of dietary replenishment. We then repeated the analysis with an expanded panel of bacterial strains and performed a metagenome-wide association (MGWA) study to identify distinct bacterial genes that are significantly correlated with the level of colonization by persistent bacterial strains. Based on the MGWA study, we tested if 44 bacterial transposon insertion mutants from 6 gene categories affect bacterial persistence within the flies. We identified that transposon insertions in four flagellar genes, one urea carboxylase gene, one phosphatidylinositol gene, one bacterial secretion gene, and one antimicrobial peptide (AMP) resistance gene each significantly influenced the colonization of D. melanogaster by an Acetobacter fabarum strain. Follow-up experiments revealed that each flagellar mutant was nonmotile, even though the wild-type strain was motile. Taken together, these results reveal that transposon insertions in specific bacterial genes, including motility genes, are necessary for at least one member of the fly microbiota to persistently colonize the fly.

**IMPORTANCE** Despite the growing body of research on the microbiota, the mechanisms by which the microbiota colonizes a host can still be further elucidated. This study identifies bacterial genes that are associated with the colonization persistence phenotype of the microbiota in Drosophila melanogaster, which reveals specific bacterial factors that influence the establishment of the microbiota within its host. The identification of specific genes that affect persistence can help inform how the microbiota colonizes a host. Furthermore, a deeper understanding of the genetic mechanisms of the establishment of the microbiota could aid in the further development of the *Drosophila* microbiota as a model for microbiome research.

## INTRODUCTION

Drosophila melanogaster is one of the best-studied genetic models in existence. The genetics of D. melanogaster have been researched for over a century, and the understanding of the genetics of D. melanogaster is thorough and expansive (1). One recent area of study that has gained attention in relation to D. melanogaster is that of microbiome studies. In D. melanogaster, the microbiota can influence diverse D. melanogaster phenotypes and behaviors (2–4), including life history traits like fecundity (5), life span (6), and starvation resistance (7). The D. melanogaster gut microbiota resides primarily in the foregut and crop of the fly and is well characterized, usually comprising fewer than 100 bacterial taxa and numerically dominated by fewer than 10 taxa, which are usually from the acetic acid bacteria, lactic acid bacteria, and enterobacteria (2, 8–10). Microbial diversity in laboratory flies can be further reduced, where in an extreme case, one species represented 92% of the community (9). Thus, the D. melanogaster microbiota is simpler than that of vertebrates colonized by hundreds of taxonomically diverse taxa (11, 12). Also, the D. melanogaster microbiota is readily manipulated under laboratory conditions. Large numbers of flies can be made axenic or mono- or polyassociated with specific bacterial strains, enabling high reproducibility, and the techniques are relatively straightforward and have low financial costs (13). The expansive body of established research on D. melanogaster genetics and microbiota composition further ensures ready access to genetic resources, strain collections, and other tools to facilitate the exploration of key biological questions (14).

The mechanisms and processes by which the microbiota colonizes D. melanogaster remain incompletely defined. Environmental bacteria gain access to the fly gut via horizontal transfer when the fly eats. An important early study suggested that the microbiota is established as flies ingest microbes in their diet and that the microbial community is thereafter maintained by continuous consumption in the diet (15). Food travels through the entirety of the D. melanogaster gut in less than an hour (16), and early work suggested that the microbiota was present as part of the bulk flow of food during this short transit time (14). At the same time, a separate study showed that the identity and abundance of the fly microbiota are inconstant within and across generations (17). Taken together, the primary conclusion was that the microbiota of D. melanogaster is transient and does not colonize the fly gut.

Later work studying the colonization of the D. melanogaster gut has refined this early view. It is now understood that some bacterial strains colonize their hosts, while others do not, and that bacteria from wild flies generally colonize their hosts better than congeneric laboratory strains of bacteria. Some of these works show that bacterial isolates can proliferate in the fly gut, allowing a stable association with the host independent of continuous uptake through diet (2, 18, 19). Specific bacterial genes that might be responsible for colonization processes were suggested by an analysis that showed that uric acid degradation and flagellar genes are present primarily in bacteria isolated from wild, but not laboratory, D. melanogaster lines (20). The main location of bacterial colonization is likely the foregut because this gut region bears the highest bacterial loads, and bacterial colonization is readily visualized within this structure (2, 8, 10). Additionally, analyses of the microbiomes of flies and their diets showed that the bacterial identities and abundances in the fly diet do not necessarily reflect the bacterial content and abundance in the host (21, 22). Taken together, these more recent findings have suggested that host factors are not solely responsible for the establishment of the microbiota in the host and that microbial factors also play key but incompletely defined roles in the process of host colonization.

The goal of this study was to identify microbial factors that affect bacterial persistence in the host D. melanogaster. To test if specific bacterial genes influence bacterial persistence within the host, we asked 3 questions: (i) Is there a significant difference in bacterial persistence levels between bacterial strains? (ii) Can differences in bacterial persistence be correlated with the presence of specific bacterial genes in the colonizing or noncolonizing bacterial strains? (iii) Can a mutant analysis confirm the associations predicted by answering the second question? Using a high-throughput assay involving serial transfers of flies onto sterile food, a metagenome-wide association (MGWA) study, and functional analysis of bacterial transposon insertion mutants, we identified transposon insertions in four flagellar genes, one urea carboxylase gene, one phosphatidylinositol gene, one bacterial secretion gene, and one antimicrobial peptide (AMP) resistance gene, each of which significantly affected the ability of Acetobacter fabarum to persist within D. melanogaster. These transposon insertions implicate specific microbial factors as being necessary for bacteria to persist within D. melanogaster.

## RESULTS

### There is species-specific variation in bacterial persistence within the host.

**FIG 1:**
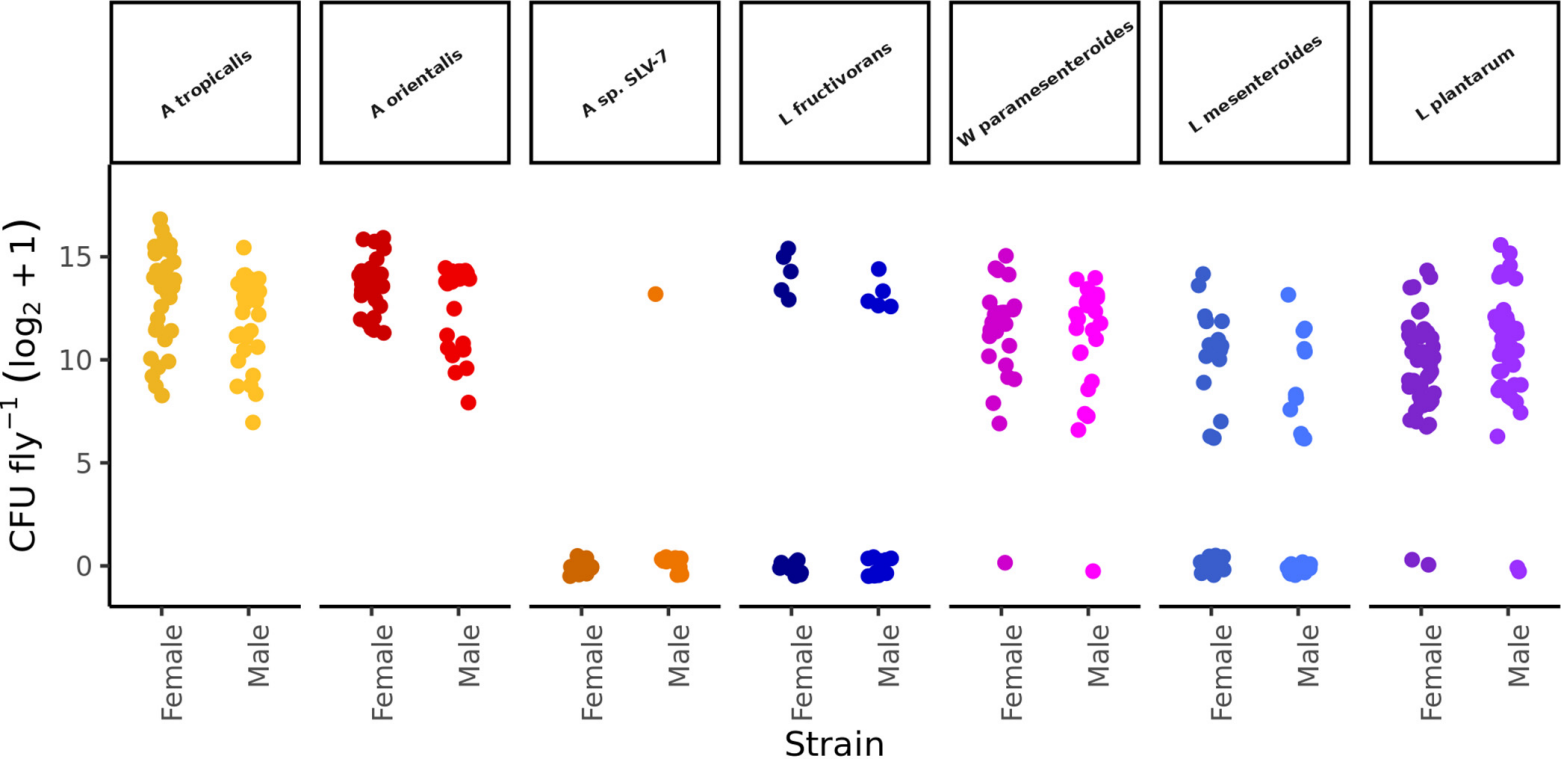
Bacterial persistence within the flies is strain specific. Each point represents the log_2_+1-transformed CFU counts for a single fly.

To begin, we developed a high-throughput assay to test the colonization persistence phenotype of a taxonomically diverse panel of bacterial strains in the flies. Our goal was to identify a regime of serial transfers that would allow us to measure bacterial persistence across a range from highly proficient to completely deficient. We detected a wide range of CFU in flies that were colonized from birth with a preliminary panel of seven bacterial strains and then, when they were 3-day-old-adults, serially transferred to sterile diets six times in total over 2 days (Fig. 1; see also Table S1 in the supplemental material). The abundances of bacteria in male and female flies reared with the seven bacterial strains from three different families varied from 0 to >88,000 CFU per fly (Kruskal-Wallis [KW] χ^2^_13; 380_ = 191.09; *P* < 1 × 10^−15^). The finding that male and female flies bore comparable bacterial loads following frequent transfer to sterile diets suggests that the persistent microorganisms may occupy similar spaces and niches between the two sexes, at least for these tested strains. We focused on just one sex in our subsequent assays and elected to study female flies, in part to make our results comparable to those of other analyses that measured life history traits of monoassociated female flies (5–7). Overall, these results confirm that our assay allowed us to detect a range of phenotypes of persistence by different bacterial strains that did not vary significantly with the sex of the flies.

### Flagellar motility is among the pathways significantly associated with bacterial persistence.

**FIG 2:**
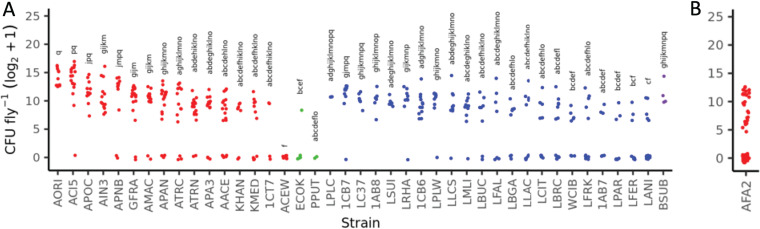
Individual data points show strain-specific differences in the bacterial persistence of 41 different bacterial strains. Each point represents the log_2_+1-transformed CFU counts from a single female fly monoassociated with different bacterial strains. The x-axis codes correspond to bacterial strains listed in Table 3. Shading matches bacterial groups: acetic acid bacteria (red), gammaproteobacteria (green), lactic acid bacteria (blue), and non-lactic acid *Firmicutes* (purple). Letters above each strain show significance groups determined by a Kruskal-Wallis test followed by Dunn’s *post hoc* test. (A) Individual data points for 41 strains in the same experiment. (B) Individual data points showing CFU of *A. fabarum*, done in a separate experiment and used for mutant testing.

To predict bacterial genes that contribute to bacterial persistence in the flies, we measured the CFU of 41 different bacterial strains in the flies after six serial transfers and statistically associated the bacterial loads with bacterial gene presence/absence patterns. The 41 different strains showed a wide range of CFU in the flies (KW χ^2^_6; 380_ = 176.04; *P* < 1 × 10^−15^), providing excellent strain-level phenotypic variation (Fig. 2; Table S2). Next, we performed an MGWA study to identify bacterial genes whose presence was associated with this variation in CFU counts. We measured the association between bacterial persistence and 12,105 orthologous groups (OGs) that were collectively spread across 3,760 phylogenetic distribution groups (PDGs) (a unique set of taxa in which an OG is present). We determined that the presence/absence patterns of 385 OGs were statistically associated with bacterial persistence in the flies (Bonferroni-corrected *P* value of <0.01) (Table S3).

From the OGs that were significantly associated with changes in bacterial persistence, we selected a subset to focus on in a mutant analysis. We chose mutants that we could obtain from an existing library of mapped and arrayed A. fabarum transposon insertion mutants (23) and that corresponded to genes in pathways that were enriched among the top MGWA hits. We assigned the 385 significant KEGG identifiers from the MGWA study to KEGG pathways and performed an enrichment analysis, which revealed that the members of the top KEGG matches were enriched for 10 KEGG pathways (Table 1; for complete results, see Table S4 in the supplemental material). Of these, we selected 26 different genes from 4 KEGG pathways for further analysis: the phosphatidylinositol signaling system, the bacterial secretion system, nicotinate and nicotinamide metabolism, and cationic antimicrobial peptide (CAMP) resistance. Among the bacterial secretion system genes were genes encoding the flagellar apparatus, which was consistent with a previous suggestion that flagellar genes were likely involved in bacterial persistence within the flies (20). From these candidates, we selected 44 mutants from five functional pathways for follow-up mutant analysis (Table 2).

**TABLE 1:** Significant results in KEGG pathway enrichment analysis

Pathway	Map ID	*P* value
Staphylococcus aureus infection	map05150	2.22E−06
Biofilm formation—Pseudomonas aeruginosa	map02025	4.11E−06
d-Alanine metabolism	map00473	4.92E−05
Nicotinate and nicotinamide metabolism	map00760	0.0005
Bacterial secretion system	map03070	0.002
Phosphatidylinositol signaling system	map04070	0.03
Atrazine degradation	map00791	0.03
Cationic antimicrobial peptide	map01503	0.044
Vancomycin resistance	map01502	0.046
Arginine biosynthesis	map00220	0.0499

**TABLE 2:** Bacterial mutants used in this study

Strain code	Gene and/or description	KEGG pathway	*P* value^*a*^
Urea carboxylase			
UCA1^*c*^	E6.3.4.6; urea carboxylase (EC 6.3.4.6)	K01941	<10^−5^
UCA2^*b*^^,^^*c*^	E6.3.4.6; urea carboxylase (EC 6.3.4.6)	K01941	<10^−5^
Phosphatidylinositol signaling			
suhB1	E3.1.3.25; *myo*-inositol-1 (or -4)-monophosphatase (EC 3.1.3.25)	K01092	0.002
suhB2^*b*^	E3.1.3.25; *myo*-inositol-1 (or -4)-monophosphatase (EC 3.1.3.25)	K01092	0.002
Bacterial secretion			
secB^*b*^	6666666.222946.peg.857 *secB*; preprotein translocase subunit SecB	K03071	0.04
yajC1	*yajC*; preprotein translocase subunit YajC	K03210	0.02
yajC2	*yajC*; preprotein translocase subunit YajC	K03210	0.02
tolC1	*tolC*; outer membrane protein	K12340	0.02
tolC2	*tolC*; outer membrane protein	K12340	0.02
Nicotinic acid			
pncB1	*pncB*; nicotinate phosphoribosyltransferase (EC 6.3.4.21)	K00763	0.02
pncB2	*pncB*; nicotinate phosphoribosyltransferase (EC 6.3.4.21)	K00763	0.02
pncC1	*pncC*; nicotinamide-nucleotide amidase (EC 3.5.1.42)	K03743	1
pncC2	*pncC*; nicotinamide-nucleotide amidase (EC 3.5.1.42)	K03743	1
pnuC1	Nicotinamide transporter, PnuC-like	K03811	<10^−5^
pnuC2	Nicotinamide transporter, PnuC-like	K03811	<10^−5^
nadC1	*nadC*; nicotinate-nucleotide pyrophosphorylase (carboxylating) (EC 2.4.2.19)	K00767	0.02
nadC2	*nadC*; nicotinate-nucleotide pyrophosphorylase (carboxylating) (EC 2.4.2.19)	K00767	0.02
nadD	*nadD*; nicotinate-nucleotide adenylyltransferase (EC 2.7.7.18)	K00969	1
AMP resistance			
degP1	*degP*; serine protease Do (EC 3.4.21.107)	K04771	0.02
degP2	*degP*; serine protease Do (EC 3.4.21.107)	K04771	0.02
degP3	*degP*; serine protease Do (EC 3.4.21.107)	K04771	0.02
degP4	*degP*; serine protease Do (EC 3.4.21.107)	K04771	0.02
degP5	*degP*; serine protease Do (EC 3.4.21.107)	K04771	0.02
degP6	*degP*; serine protease Do (EC 3.4.21.107)	K04771	0.02
mprF1^*b*^	*mprF*; phosphatidylglycerol lysyltransferase (EC 2.3.2.3)	K14205	0.04
mprF2	*mprF*; phosphatidylglycerol lysyltransferase (EC 2.3.2.3)	K14205	0.04
amiABC1	*N*-Acetylmuramoyl-l-alanine amidase (EC 3.5.1.28)	K01448	<10^−5^
amiABC2	*N*-Acetylmuramoyl-l-alanine amidase (EC 3.5.1.28)	K01448	<10^−5^
Flagellar assembly			
fliP	*fliP*; flagellar biosynthetic protein FliP	K02419	0.42
fliM1	*fliM*; flagellar motor switch protein FliM	K02416	1
fliM2	*fliM*; flagellar motor switch protein FliM	K02416	1
fliH1	*fliH*; flagellar assembly protein FliH	K02411	1
fliH2	*fliH*; flagellar assembly protein FliH	K02411	1
fliG1	*fliG*; flagellar motor switch protein FliG	K02410	1
fliG2	*fliG*; flagellar motor switch protein FliG	K02410	1
fliF1^*b*^^,^^*c*^	*fliF*; flagellar M-ring protein FliF	K02409	1
fliF2^*b*^^,^^*c*^	*fliF*; flagellar M-ring protein FliF	K02409	1
fliI1^*c*^	*fliI*; flagellum-specific ATP synthase (EC 3.6.3.14)	K02412	1
fliI2^*b*^^,^^*c*^	*fliI*; flagellum-specific ATP synthase (EC 3.6.3.14)	K02412	1
flgK1	*flgK*; flagellar hook-associated protein 1 FlgK	K02396	1
flgK2	*flgK*; flagellar hook-associated protein 1 FlgK	K02396	1
flgE1^*b*^^,^^*c*^	*flgE*; flagellar hook protein FlgE	K02390	1
flgE2^*c*^	*flgE*; flagellar hook protein FlgE	K02390	1
flgF1	*flgF*; flagellar basal body rod protein FlgF	K02391	1
flgF2	*flgF*; flagellar basal body rod protein FlgF	K02391	1
flgH1^*b*^^,^^*c*^	*flgH*; flagellar L-ring protein precursor FlgH	K02393	1
flgH2^*b*^^,^^*c*^	*flgH*; flagellar L-ring protein precursor FlgH	K02393	1
flgI1	*flgI*; flagellar P-ring protein precursor FlgI	K02394	1
flgI2	*flgI*; flagellar P-ring protein precursor FlgI	K02394	1
flgB	*flgB*; flagellar basal body rod protein FlgB	K02387	1
flhB1	*flhB*; flagellar biosynthetic protein FlhB	K02401	1
flhB2	*flhB*; flagellar biosynthetic protein FlhB	K02401	1
flgA1	*flgA*; flagellar basal body P-ring formation protein FlgA	K02386	1
flgA2	*flgA*; flagellar basal body P-ring formation protein FlgA	K02386	1

a Bonferroni-corrected *P* value from the MGWA study.

b Persistence phenotype significantly different from that of the wild type.

c Tested for motility.

### Flagellar motility genes are among those that influence bacterial persistence within the flies.

**FIG 3:**
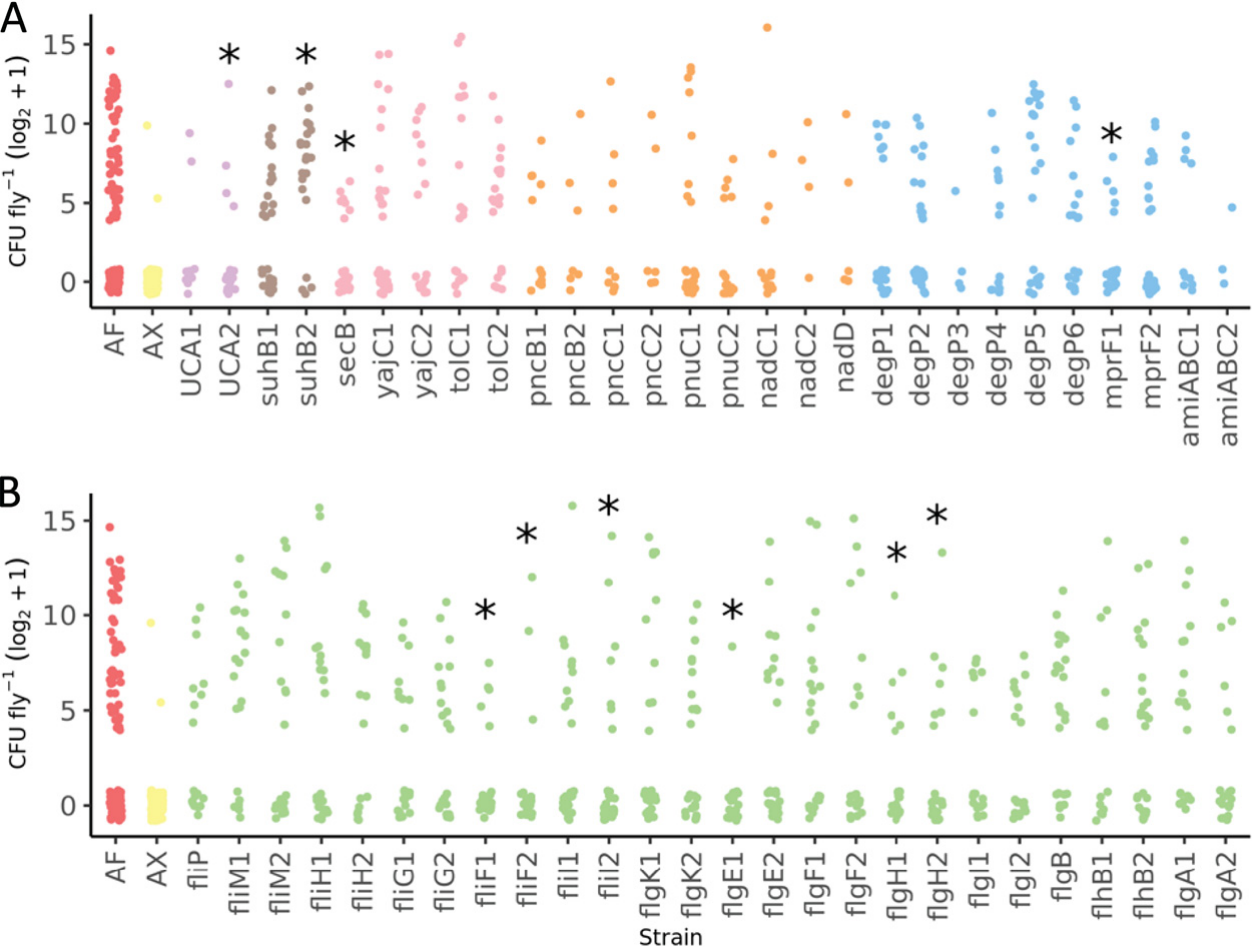
Transposon insertion mutants affect the bacterial persistence phenotype. Shown are individual data points of the log_2_+1-transformed CFU per fly for all mutants tested in the assay, with each point representing a single female fly. The x-axis codes correspond to transposon insertion mutants listed in Table 2. Shading matches the type of mutants tested: wild-type *A. fabarum* [AF] (red), bacterium-free (axenic [AX]) flies (yellow), urea carboxylase (purple), phosphatidylinositol (brown), bacterial secretion (pink), nicotinate metabolism (orange), AMP resistance (blue), and flagellar motility (green). Asterisks indicate that the bacterial mutants had a persistence phenotype that was significantly different from that of the wild-type bacterial strain (AF), determined by pairwise Kruskal-Wallis tests (*P* < 0.05).

We determined the persistence phenotype of bacterial mutants for genes identified in the MGWA study as a step toward validating MGWA predictions and identifying bacterial gene candidates that influence bacterial persistence within the flies. We identified 10 transposon insertions mutants in 8 different genes from 5 different gene categories that significantly influenced bacterial persistence within the flies (KW χ^2^_56; 1,239_ = 189.32; *P* < 10^−15^) (Fig. 3; Table S5). Nine of the mutants conferred a lower-persistence phenotype, and one mutant, a *myo*-inositol-monophosphatase mutant, conferred a higher-persistence phenotype. The identification of some mutants with either greater or poorer persistence than the wild-type strain of *A. fabarum* highlights the benefit of using a bacterial strain with an intermediate persistence phenotype for mutant analyses. Six of the significant mutants were flagellar assembly mutants, representing four flagellar genes. These efforts confirm that multiple different bacterial pathways influence bacterial persistence within the flies and especially implicate bacterial flagellar genes as possible effectors of this phenotype.

### Flagellar mutants that persist poorly within the flies are nonmotile.

**FIG 4:**
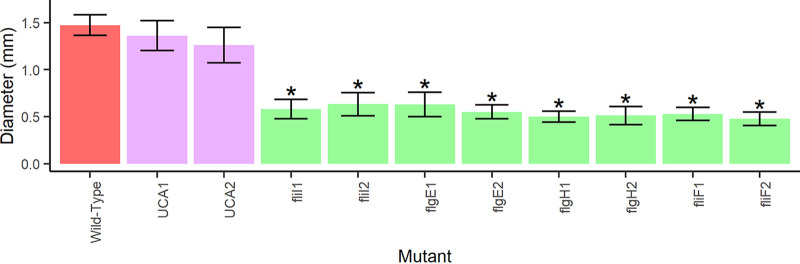
Flagellar mutants that influence persistence are nonmotile. The mean diameters measured for the halos from the motility tests for each bacterial strain are shown. Shading matches the type of mutants tested: wild-type *A. fabarum* (red), urea carboxylase (purple), and flagellar assembly (green). Asterisks are shown if the average diameter of a mutant strain differed significantly from that of the wild-type strain. Significant differences between diameters were determined by a linear mixed-effects model with a Dunnett *post hoc* test applied.

To determine if bacterial motility is associated with bacterial persistence, we compared the motility phenotypes of flagellar mutants, nonflagellar mutants, and wild-type *A. fabarum*. All flagellar mutants that were tested were nonmotile, while wild-type *A. fabarum* and the nonflagellar mutants were all motile (*P* < 5.176 × 10^−15^ by analysis of variance [ANOVA] [linear mixed model]; *f* = 13.662) (Fig. 4; Table S6). The correlation between flagellar mutants involved in bacterial persistence and nonmotility indicates that bacterial motility may play a role in bacterial persistence in flies.

## DISCUSSION

This work adds to the growing knowledge of how the microbiota is established and persists within D. melanogaster. The identification of bacterial genes that are involved in bacterial persistence within the flies shows that the host alone is not responsible for the establishment and persistence of the gut microbiota. In particular, we identified that transposon insertions in four flagellar genes, one urea carboxylase gene, one phosphatidylinositol gene, one bacterial secretion gene, and one AMP resistance gene affect the bacterial persistence of a representative member of the gut microbiota, *A. fabarum*. These findings identify candidate pathways and mechanisms that underlie the bacterial persistence of the microbiota.

Here, we reveal that transposon insertions in four flagellar genes affect bacterial persistence within the flies. Motility is associated with the function of these flagellar genes because each persistence-deficient flagellar mutant was also nonmotile, suggesting that motility contributes to bacterial persistence in the flies. We have not defined any specific roles for motility in bacterial persistence within the flies, but a possible explanation could be that motility enables bacteria to find favorable niches through bacterial taxis within the *Drosophila* foregut, particularly in the proventriculus region, which has been shown to have a microbial niche (10). A favorable niche could be a region of the fly that has a better quantity or quality of nutrients, less immune stress, or decreased competition with other microorganisms. Also, other studies have shown that bacteria with intact, motile flagella are at an advantage for host colonization (24–26). Alternatively, these flagellar genes may be involved in bacterial secretion (27). Bacterial secretion could create a favorable environment or be beneficial for the host. Bacterial surface components, including flagella, are also known to interact directly with the gut epithelium, which could also promote persistence (28). Counter to either of these expectations is the finding that some high-persisting bacterial strains like Acetobacter pomorum DmCS_004 lack flagellar motility genes (see Table S4 in the supplemental material). Therefore, if motility is necessary for colonization, then A. pomorum must do so independently of the prototypical flagellar machinery, or motility may be one of several redundant mechanisms that contribute to bacterial persistence within the host.

In addition to flagellar motility genes, we identified transposon insertions in other genes that were significantly associated with variation in bacterial persistence within the flies. One of these is in an AMP resistance gene, the flippase gene *mprF*, which translocates lysyl-phosphatidylglycerol to the outer leaflet of the membrane. It is also involved in resistance to multiple antimicrobial peptides from the host and other competing microorganisms (29). AMP resistance has already been identified as a mechanism for the stable association of gut commensals in the human gut during periods of host inflammation (30). Pathogens are also able to stably colonize the gut due to AMP resistance (31), so it is possible that beneficial microbes also stably colonize the gut through AMP resistance. Some AMP resistance genes such as *degP* (although not significant in our analysis) have been shown to help bacteria adjust to survive at high temperatures by decreasing temperature-sensitive growth (32). AMP resistance in bacteria may be important because AMPs were shown to be necessary for the regulation of the microbiota abundance in the gut of D. melanogaster (33). Overall, AMP resistance can help microbes colonize the gut by protecting against host antimicrobial peptides and helping the bacteria to adjust to a changing environment.

Of the 3 bacterial secretion genes that we tested, only transposon insertions in *secB*, which is involved in the quorum sensing, protein export, and bacterial secretion system pathways, significantly affected bacterial persistence within the flies. *secB* specifically exports proteins but is also involved in stress-responsive type II toxin-antitoxin (TA) systems (34), which have been shown to be important for niche-specific colonization by Escherichia coli in humans (35). Another gene, *yajC*, is also involved in these pathways but was not shown to be significant. The identification of some but not all genes in a pathway as being significant for bacterial persistence leads to the idea that only particular parts of the pathways are important for the bacterial persistence phenotype. The *sec* bacterial secretion system is used by pathogenic bacteria to secrete virulence factors (36), hinting at a possible interaction with the host. Bacterial secretion can also aid in establishing a hospitable niche for the bacteria by the secretion of products such as AMP resistance products, which are known substrates of SecB (37).

The other two transposon insertions that significantly affected bacterial persistence within the flies were in urea carboxylase and *myo*-inositol-1 (or -4)-monophosphatase genes. Urea carboxylase is involved in arginine biosynthesis, atrazine degradation, and metabolic pathways (38, 39). *Myo*-Inositol-1 (or -4)-monophosphatase is involved in streptomycin biosynthesis, inositol phosphate metabolism, metabolic pathways, the biosynthesis of secondary metabolites, and the phosphatidylinositol signaling system (40–43). Since these genes are not closely related to other significant genes and are involved in many different pathways, further research would be required to understand the role that they play in bacterial persistence.

### Future directions.

In conclusion, we show here that transposon insertions in bacterial genes, particularly flagellar genes, are implicated in bacterial persistence in flies, and we advocate for the study of the role of flagellar genes in the colonization of flies and other organisms. Flagellar genes have been shown to play a role in the colonization and pathogenicity of Listeria monocytogenes in plants (25), Salmonella enterica serovar Enteritidis (24) and Campylobacter jejuni (26) in chickens, and Escherichia coli in mice and humans (44, 45). Our data suggest that motility is a likely role for the flagellar genes in the host colonization phenotype that we describe, but other flagellar machinery functions, such as bacterial secretion and bacterial adhesion, can also have important roles in host-microbe interactions (46). Due to the diverse functions of flagella, further investigation into how flagella influence and permit host colonization is likely to reveal additional insights into how bacteria can colonize and persist within their animal hosts.

## MATERIALS AND METHODS

### Bacterial and fly cultures.

The fly stock was originally obtained from Mariana Wolfner at Cornell University and is a *Wolbachia*-free stock of Canton-S D. melanogaster flies. The stock flies were raised in an incubator with a 12-h-light/12-h-dark cycle at 25°C. They were raised on a yeast-glucose (YG) diet that contains 10% brewer’s yeast, 10% glucose, 1% agar, 0.084% propionic acid, and 0.08% phosphoric acid.

Stocks of bacterial strains were stored at −80°C. The bacterial strains were streaked for isolation onto clade-specific medium plates and incubated at 30°C for 2 to 3 days (Table 3). The different medium types were modified deMan, Rogosa, and Sharpe (mMRS) (catalog number C5932; Criterion), lysogeny broth (LB) (catalog number 11-119; Apex), and potato dextrose (catalog number 70139-500G; Sigma-Aldrich). Aerobic strains were placed in an incubator, while anaerobic strains were put into carbon dioxide-flooded containers that were sealed and placed in an incubator. One colony was then removed from the plates, placed into a tube of 5 mL of clade-specific medium broth, and incubated at 30°C for 1 to 2 days. If the strains were aerobic, the tubes of liquid broth were grown under oxic conditions by shaking. Aerotolerant strains were raised under microoxic conditions by remaining static. The bacteria were then diluted at a 1:8 dilution four times and normalized to an optical density at 600 nm (OD_600_) of 0.01.

**TABLE 3:** Bacterial strains used in this study^*a*^

Strain	Strain code^*b*^	Medium	WGS ID
Acetobacter aceti NBRC 14818	AACE	MRS	SLZP01
*Acetobacter* sp. strain DsW_054	AFA2	MRS	JOPD01
Acetobacter indonesiensis DmW_046	AIN3	MRS	JOMP01
Acetobacter malorum DmCS_005	AMAC	MRS	JOJU01
Acetobacter orientalis DmW_045	AORI	MRS	JOMO01
Acetobacter orientalis DmW_048	ACEW	MRS	JOOY01
Acetobacter pasteurianus 3p3	APA3	MRS	CADQ01
Acetobacter pasteurianus NBRC 101655	APAN	MRS	AP014881.1
Acetobacter pasteurianus NBRC 106471 or LMG1262^T^	APNB	MRS	BDER01
Acetobacter pomorum DmCS_004	APOC	MRS	JOKL01
*Acetobacter* sp. strain DmW_043	ACI5	MRS	JOMN01
*Acetobacter* sp. strain SLV-7 DmW_125	1CT7	MRS	VHOZ01
Acetobacter tropicalis DmCS_006	ATRC	MRS	JOKM01
Acetobacter tropicalis NBRC 101654	ATRN	MRS	BABS01
Bacillus subtilis strain 168	BSUB	LB	NC_000964.3
Escherichia coli strain K-12 substrain MG1655	ECOK	LB	NC_000913.3
Fructilactobacillus fructivorans KCTC 3543	LFRK	MRS	QSLO01
Gluconobacter frateurii NBRC 101659	GFRA	POT	BADZ02
Novacetimonas hansenii ATCC 23769	KHAN	POT	ADTV01
Komagataeibacter medellinensis NBRC 3288	KMED	POT	NC_016037.1
*Lacticaseibacillus paracasei* DmW 181	LPAR	MRS	NDXH01
*Lacticaseibacillus rhamnosus* GG	LRHA	MRS	NC_013198.1
*Lactiplantibacillus plantarum* DmCS_001	LPLC	MRS	JOJT01
*Lactiplantibacillus plantarum* WCFS1	LPLW	MRS	NC_004567.2
*Lentilactobacillus hilgardii* ATCC 27305	LBGA	MRS	NZ_GG669709.1
Lentilactobacillus buchneri NRRL B-30929	LBUC	MRS	NC_015428.1
Lactococcus lactis BPL1	LLAC	MRS	JRFX01
Lactococcus lactis DmW198	LLCS	MRS	NEQN01
Leuconostoc citreum DmW_111	LCIT	MRS	NDXG01
Leuconostoc citreum DmW_137	LC37	MRS	JADAXK01
Leuconostoc fallax KCTC 3537	LFAL	MRS	AEIZ01
Fructilactobacillus fructivorans DMCS_002	LFRC	MRS	JOJZ01
Leuconostoc suionicum DmW_098	LSUI	MRS	JADAXL01
Levilactobacillus brevis DmCS_003	LBRC	MRS	JOKA01
*Ligilactobacillus animalis* KCTC 3501 DSM 20602	LANI	MRS	AYYW01
*Limosilactobacillus fermentum* ATCC 14931	LFER	MRS	ACGI01
*Liquorilactobacillus mali* KCTC 3596 (DSM 20444)	LMLI	MRS	AYYH01
Pseudomonas putida F1	PPUT	LB	NC_009512.1
Weissella cibaria DmW_103	WCIB	MRS	NDXJ01
Weissella paramesenteroides DmW_107	1CB6	MRS	VHPP01
Weissella paramesenteroides DmW_109	1AB8	MRS	VHPI01
Weissella paramesenteroides DmW_115	1CB7	MRS	VHPE01
Weissella paramesenteroides DmW_118	1AB7	MRS	VHPB01
Weissella paramesenteroides DmW_107100	1CB3	MRS	VHPR01

a WGS, whole-genome sequencing; POT, potato dextrose medium.

b The strain abbreviations are used in Fig. 2.

### Axenic and monoassociated flies.

All flies used in the persistence assay were derived as bacterium-free embryos before they were inoculated with bacteria. Fly eggs were made axenic by removing the chorion layer of the eggs. To do this, stock flies were allowed to lay eggs for 18 to 20 h on a plate containing 10% brewer’s yeast, 10% glucose, 1% agar, and grape juice. The eggs were then collected and washed with a 0.6% hypochlorite solution twice for 2.5 min each. They were then washed three times with double-distilled, autoclaved water. Next, 40 to 60 eggs were transferred into 50-mL vials containing 7.5 mL of the autoclaved YG diet.

To monoassociate the flies, 50 μL of normalized bacteria was inoculated into axenic eggs with the sterile diet. The fly vials were then placed on a tray and put into an incubator at 25°C with a 12-h-light/12-h-dark cycle.

### Persistence assay.

Bacterial persistence within the flies was measured using an assay that frequently transfers adult flies to a sterile diet. Four days after bulk eclosion of the flies, 4 female flies from each vial were transferred under carbon dioxide anesthesia into separate wells of a 96-well plate with 150 μL of the sterile diet at the bottom. The flies were then transferred to new 96-well plates containing sterile food 3 times a day (8 a.m., 1 p.m., and 6 p.m.) for 2 days. After the last transfer, the flies were placed into 1.7-mL microcentrifuge tubes with 150 μL of phosphate-buffered saline (PBS) and 150 μL of ceramic beads and homogenized in a GenoGrinder instrument for 2 min at 1,750 rpm. The contents of the microcentrifuge tubes were then dilution plated and cultured in an incubator at 30°C until the colonies were large enough to count (around 2 to 3 days); each colony was counted as 1 CFU and used as a measure of persistence. If the colonies were too dense to count, then 160 was used as the CFU number, corresponding to 128,000 CFU per fly. The first analysis was performed with 7 strains, and a Kruskal-Wallis test and pairwise Wilcoxon tests between sexes for each strain were performed to assess if the CFU per fly were significantly different. The second analysis was performed with 41 different strains for use in a metagenome-wide association (MGWA) study, and a Kruskal-Wallis test with Dunn’s *post hoc* test was performed to determine significance groups for CFU per fly among all strains. The third analysis was performed with 44 mutants identified by the MGWA study, and a Kruskal-Wallis test was performed to test if each mutant was significantly different from the wild-type control. In each experiment, each treatment had triplicate vials in each of three separate experiments. Vials were discarded from the analysis if they were contaminated or the vial density was <30 flies. A vial was determined to be contaminated if undiluted aliquots bore more than 5 CFU of an unexpected colony morphology.

### Metagenome-wide association.

An MGWA study was performed to predict bacterial genes that influence persistence. In order to perform the MGWA study, amino acid sequences were obtained from GenBank for the exact strains that we phenotyped. The amino acid sequences of 55 bacterial genomes (see Table S7 in the supplemental material) were clustered into orthologous groups (OGs) using OrthoMCL (47) with an inflation factor of 1.5. The MGWA study was then performed using the R package MAGNAMWAR (48). The inputs for MAGNAMWAR were the clusters of orthologous group assignments and the CFU per fly at the end of the persistence assay. The MGWA study associated OG presence/absence patterns with bacterial persistence levels using a Wilcoxon test. The resultant *P* values were Bonferroni corrected, and we set an arbitrary significance threshold of a *P* value of <0.01. A KEGG enrichment analysis was then done to find functional categories enriched among the significant OGs. BlastKOALA (49) was used to assign KEGG functions to a representative sequence from each OG. Pathway significance was then determined by a false discovery rate (FDR)-corrected chi-square test.

### Motility assay.

A motility assay was performed by placing 1 μL of OD_600_-normalized bacteria in PBS on an mMRS plate with 2 g of agar per L, replacing the normal plates. The plates were then left at room temperature for 48 to 72 h, and the diameter of the halo that formed on the plates was measured using a ruler. The tests were performed on flagellar mutants, urea carboxylase mutants, and the wild-type strain of *A. fabarum* (Table 2) (50). A linear mixed-effects model was used to test if there was a significant effect of the bacterial genotype on the bacterial halo size, and a Dunnett *post hoc* test was then applied to determine if there were significant differences between the mean diameter of each mutant and that of the wild-type strain.
